# Vitamin D levels in children with COVID-19: a report from Turkey

**DOI:** 10.1017/S0950268821001825

**Published:** 2021-08-10

**Authors:** Aysegul Alpcan, Serkan Tursun, Yaşar Kandur

**Affiliations:** Department of Pediatrics, School of Medicine, Kirikkale University, Kirikkale, Turkey

**Keywords:** Children, COVID-19, vitamin D

## Abstract

Several studies have demonstrated that higher levels of vitamin D are associated with better prognosis and outcomes in infectious diseases. We aimed to compare the vitamin D levels of paediatric patients with mild/moderate coronavirus disease 2019 (COVID-19) disease and a healthy control group. We retrospectively reviewed the medical records of patients who were hospitalised at our university hospital with the diagnosis of COVID-19 during the period between 25 May 2020 and 24 December 2020. The mean age of the COVID-19 patients was 10.7 ± 5.5 years (range 1–18 years); 43 (57.3%) COVID-19 patients were male. The mean serum vitamin D level was significantly lower in the COVID-19 group than the control group (21.5 ± 10.0 *vs*. 28.0 ± 11.0 IU, *P* < 0.001). The proportion of patients with vitamin D deficiency was significantly higher in the COVID-19 group than the control group (44% *vs.* 17.5%, *P* < 0.001). Patients with low vitamin D levels were older than the patients with normal vitamin D levels (11.6 ± 4.9 *vs.* 6.2 ± 1.8 years, *P* = 0.016). There was a significant male preponderance in the normal vitamin D group compared with the low vitamin D group (91.7% *vs.* 50.8%, *P* = 0.03). C-reactive protein level was higher in the low vitamin D group, although the difference did not reach statistical significance (9.6 ± 2.2 *vs.* 4.5 ± 1.6 mg/l, *P* = 0.074). Our study provides an insight into the relationship between vitamin D deficiency and COVID-19 for future studies. Empiric intervention with vitamin D can be justified by low serum vitamin D levels.

## Introduction

Coronavirus disease 2019 (COVID-19) has become a public health threat to the world population during the last year. The lower airway tract is the primary target of the infection [1]. Acute respiratory distress syndrome (ARDS), septic shock and coagulation disorders are the severe complications of this infection, which are rare in children [2]. A systemic inflammatory response resulting from the release of large amounts of inflammatory cytokines leads to ARDS [3]. Although it has been stated at the beginning of the pandemic that children are affected less seriously than adults, it has been observed that children were also affected by the infection at least as severely as adults [4]. It is known that vitamin D regulates the immune response in infectious and autoimmune diseases; its immunomodulatory role has been reported in several studies. It is also known that vitamin D augments the expression of two antimicrobial peptides called cathelicidin and *β*-defensin, which play a key role in innate immunity [5, 6]. These peptides are involved in direct microbicidal effects and have also shown pleiotropic effects in inducing immunomodulatory responses to pathogenic stimuli. Vitamin D has both a direct and indirect impact on T-lymphocytes. Vitamin D downregulates type 1 T cells and upregulates type 2 T cells. It indirectly modulates the T-lymphocyte stimulatory function of antigen-presenting cells. It also facilitates the induction of T regulatory cells and inhibits IL-17 secretion by Th17 cells, and directly upregulates IL-4 in T cells [7–9].

Several studies have demonstrated that higher levels of vitamin D are associated with better prognosis and outcomes in infectious diseases [10]. In another study, Darren *et al*. studied the vitamin D level of 25 children with COVID-19. They found that 72% of the patients were vitamin D deficient. Moreover, its level was significantly lower than the control group [11]. It has been reported that the severity of respiratory tract infections is reduced by vitamin D administration [12]. In our retrospective study, we aimed to compare the vitamin D levels of paediatric patients with mild/moderate COVID-19 disease and a healthy control group.

## Methods

We retrospectively reviewed the medical records of patients who were hospitalised at our university hospital with the diagnosis of COVID-19 during the period between 25 May 2020 and 24 December 2020 after local ethical committee approval. The control group was selected from healthy children aged 1 month to 17 years who were examined at the healthy child division and whose serum vitamin D levels were measured. Serum vitamin D levels were measured in Cobas E411 device (Roche Diagnostic, Switzerland) by enzyme-linked immunosorbent assay using commercial kits (new Roche Elecsys Vitamin D Total II assay, Switzerland), according to the manufacturers’ instructions. Written informed consent was obtained from the parents for hospitalisation. The demographic characteristics, medical history, symptoms, signs and laboratory findings of the patients were collected from the patients’ medical records. Serum samples of the patients were collected at disease onset to study routine blood tests, infection biomarkers and vitamin D levels. Patients with bone metabolism disorders were excluded. The demographic characteristics and serum 25-OH vitamin D levels of the patients were recorded. Patients with 25-OH vitamin D levels below 20 ng/ml (<50 nmol/l) were considered to have vitamin D deficiency; those with 25-OH vitamin D levels between 21 and 29 ng/ml (52.5 and 72.5 nmol/l) were considered to have vitamin D insufficiency; and those with 25-OH vitamin D levels above 30 ng/ml were considered to have a normal vitamin D level [13]. Patients with a positive COVID-19 RT-PCR (reverse transcription-polymerase chain reaction) test but without clinical and radiological signs of the disease were considered asymptomatic; patients with symptoms of an upper respiratory tract infection such as fever, fatigue, myalgia, cough, sore throat and runny nose but a normal respiratory system examination were considered to have a mild disease; and patients with signs of pneumonia and the complaints of fever and cough but without symptoms of dyspnoea and hypoxemia were considered to have pneumonia. Respiratory distress was defined as having tachypnoea and the need for supplementary oxygen [14]. Asymptomatic patients were excluded. The patient and control groups were compared with respect to the vitamin D level. Additionally, COVID-19 patients with normal *vs.* low vitamin D levels were compared with regard to the clinical and laboratory variables. The ethics committee approval of the study was obtained from the Kirikkale University Clinical Research Ethics Committee (Date: 25.02.2021/Decision no:2021.02.03)

### Statistical analysis

We reported categorical variables in the form of frequency and percentage and continuous variables in the form of mean and standard deviation (s.d.). The Student's *t* test was used to compare quantitative variables in two independent groups. The *χ*^2^ test and Fisher's exact test were used to compare categorical variables between two independent groups. All statistical analyses were performed using SPSS (Statistical Package for the Social Sciences) version 20.0 software (Armonk, NY: IBM Corp., USA). A two-sided *P*-value of <0.05 was considered statistically significant. Any correlation between the laboratory variables and vitamin D level was sought using a non-parametric correlation test (Spearman's rank correlation test).

## Results

We evaluated the medical records of 75 COVID-19 patients and 80 healthy controls. The mean age of the COVID-19 patients was 10.7 ± 5.5 years (range 1–18 years); 43 (57.3%) patients were male. Seventy (92.1%) patients had a history of contact with a COVID-19-positive person. The control group was composed of 43 (53.8%) males and 37 (46.2%) females and had a mean age of 9.9 ± 4.6 years (range 1–17 years). There was no significant difference between the COVID-19 group and the control group with respect to mean age and gender distribution (Table 1). The serum vitamin D level was significantly higher in females than males (12.3 ± 4.3 *vs.* 9.6 ± 6.0; *P* = 0.028) in the COVID-19 group. The presenting symptoms included fever (*n* = 46, 61.3%), cough (*n* = 29, 38.7%), respiratory distress (*n* = 9, 12%), nasal congestion/runny nose (*n* = 5, 6.6%), headache (*n* = 9, 12%), weakness (*n* = 21, 28%) and myalgia (*n* = 9, 12%). Two (2.6%) patients had rales/rhonchi on lung auscultation. None of the patients had severe disease nor any of them was admitted to the intensive care unit. None of the patients died from the disease. The mean serum vitamin D level was significantly lower in the COVID-19 patient group than the control group (21.5 ± 10.0 *vs.* 28.0 ± 11.0 IU; *P* < 0.001). Twelve patients had a normal vitamin D level; 33 patients had vitamin D deficiency, and the rest of the patients (*n* = 30) had vitamin D insufficiency. The ratio of patients with vitamin D deficiency was significantly higher in the COVID-19 group than the control group (44% *vs.* 17.5%, *P* < 0.001) (Table 1).

**Table 1.** Comparison of patients and control groups with respect to age, gender distribution and vitamin D levels

	COVID-19 patients *N* = 75	Control *N* = 80	*P*
Mean age (years)	10.7 ± 5.5	9.9 ± 4.6	0.122
Gender (m/%)	43/57.3	43/53.8	0.654
Mean serum vitamin D (IU)	21.5 ± 10.0	28.0 ± 11.0	<0.001*
Vitamin D			
Deficiency	33 (44)	14 (17.5)	<0.001*
Insufficient	30 (40)	39 (48.8)	0.273
Normal	12 (16)	27 (33.7)	0.01*

* *p* < 0.05.

COVID-19 patients with low vitamin D levels were older than the COVID-19 patients with normal vitamin D levels (11.6 ± 4.9 *vs.* 6.2 ± 1.8 years; *P* = 0.016). There was a significant male preponderance in the normal vitamin D group compared to the low vitamin D group (91.7% *vs.* 50.8%; *P* = 0.03). Patients with low vitamin D levels had significantly lower rates of fever, cough, but higher rates of respiratory distress, weakness, anosmia, headache, myalgia and taste loss (*P* < 0.05, Table 2). The mean lymphocyte count was significantly lower in the low vitamin D group compared with the normal vitamin D group (2300 ± 1200 *vs.* 4200 ± 2900 count/μl; *P* = 0.049). However, there was no significant difference between mean levels of haemoglobin, platelet count, albumin, creatinine kinase and D-dimer (*P* > 0.05). C-reactive protein (CRP) level was higher in the low vitamin D group, although the difference did not reach statistical significance (9.6 ± 2.2 *vs.* 4.5 ± 1.6 mg/l; *P* = 0.074). Three patients in the low vitamin D group and one patient in the normal vitamin D group had moderately severe disease.

**Table 2.** Comparison of the laboratory tests and other variables between the low and normal vitamin D

Variable	Low-intermediate vitamin D level *n* = 63	Normal vitamin D level *n* = 12	*P*
Mean age (years)	11.6 ± 4.9	6.2 ± 1.8	0.016*
Gender (m/%)	32 (50.8)	11 (91.7)	0.03*
Mean serum vitamin D (IU)	18.5 ± 7.2	37.4 ± 7.3	<0.001*
Fever (*n*/%)	38 (60.3)	8 (66.7)	<0.001*
Cough (*n*/%)	23 (36.5)	6 (50)	<0.001*
Respiratory distress (*n*/%)	8 (12.7)	0	<0.001*
Cyanosis (*n*/%)	0	0	
Weakness (*n*/%)	18 (28.6)	3 (25.0)	<0.001*
Anosmia (*n*/%)	6 (9.7)	0	<0.001*
Headache (*n*/%)	8 (12.7)	1 (8.3)	<0.001*
Myalgia (*n*/%)	8 (12.7)	1 (8.3)	<0.001*
Moderately severe presentation (*n*/%)	3 (4.8)	1 (8.3)	<0.001*
Loss of taste (*n*/%)	3 (4.8)	0	
Mean leucocyte count/μl	6300 ± 2400	8900 ± 4200	0.058
Mean lymphocyte count/μl	2300 ± 1200	4200 ± 2900	0.049*
Mean eosinophil count/μl	99 ± 18	143 ± 58	0.483
Mean haemoglobin (g/dl)	13.2 ± 1.1	12.6 ± 14.0	0.163
Mean platelet count/μl	312 000 ± 92 000	259 000 ± 74 000	0.083
Mean CRP (mg/l)	9.6 ± 2.2	4.5 ± 1.6	0.074
Mean creatinine (mg/dl)	0.6 ± 0.1	0.5 ± 0.1	0.029*
Mean albumin (gr/dl)	4.4 ± 0.4	4.3 ± 0.3	0.179
Mean CK (U/l)	150 ± 47	397 ± 228	0.312
Mean D-dimer (ng/ml)	223 ± 48	296 ± 106	0.540
PA X-ray findings	8 (12.7)	0	<0.001*

* *p* < 0.05.

Correlation analysis showed a positive correlation between vitamin D level and leukocyte count, lymphocyte count and platelet count; and a negative correlation with age and the duration of hospitalisation (Table 3). There was no correlation between CRP and vitamin D level (*r* = −0.057, *P* = 0.627) or body temperature and vitamin D level (*r* = −0.116, *P* = 0.323).

**Table 3.** Variables that were significantly correlated to serum vitamin D level

	Vitamin D level
Age	*P* < 0.001 *r* = −0.385
Leucocyte count	*P* = 0.008 *r* = 0.306
Lymphocyte count	*P* < 0.001 *r* = 0.437
Platelet count	*P* = 0.002 *r* = 0.345
Duration of hospitalisation	*P* = 0.005 *r* = −0.724

The regression analysis showed that low vitamin D level was a risk factor for the occurrence of respiratory distress (OR −0.268, 95% CI −15.920 to −1.406; *P* = 0.02). However, it was not a risk factor for fever, high CRP, myalgia, headache and the duration of hospitalisation.

## Discussion

To our knowledge, this is one of the primary studies that analysed the relationship between vitamin D level and COVID-19 infection in a paediatric age group; however, our study had a larger sample size. Our study revealed that vitamin D deficiency is correlated to COVID-19 severity.

Children constitute about 1% of all COVID-19 cases in Turkey [4]. COVID-19 has a milder course in children than adults. Previous reports have shown that the proportion of children with high levels of inflammatory markers is low [15]. Our short-time experience with COVID-19 has shown that symptoms are less severe in children than adults [16]. In the present study, we reported the clinical manifestations and serum vitamin D levels of 75 Turkish children with PCR-positive COVID-19.

There was a negative correlation between age and vitamin D level in the patient group. The mean age of the patients with a normal vitamin D level was higher than the low vitamin D group. This is perhaps caused by the parental behaviour in our society, such that parents continue to administer vitamin preparations even after 2 years of age. Thus, we suggest that there was a male preponderance in the normal vitamin D group.

In a similar study, Yilmaz and Şen [17] investigated the correlation between vitamin D level and COVID-19 in a paediatric age group. They also showed that vitamin D level was significantly lower in COVID-19 patients than in the controls. Akoğlu *et al*. [18] also found that the 25-hydroxyvitamin D3 (vitamin D3) level was significantly lower in the moderately severe disease group than the mild disease group (*P* = 0.044). Yilmaz and Şen reported that fever was more frequent in the vitamin D-deficient group than the normal vitamin D group; there was even no fever in patients with normal vitamin D levels. In addition, they found a negative correlation between fever and vitamin D levels. However, our results showed that fever was more frequent in patients with a normal vitamin D level. We found similar results regarding the complaint of cough. So, we considered that there may be a relationship between vitamin D level and inflammatory process, and cytokine release, which leads to fever. An adequate vitamin D level may be probably essential for the development of fever and cough. Previous studies reported that prostaglandin (PG) E is an important molecule in the pathogenesis of fever and is modulated by vitamin D [19]. This could be probably the same for cough as a previous report has shown that PG enhances cough reflex [20]; thus, vitamin D plays a role in the synthesis of PG.

We found a lower, albeit statistically non-significant, CRP elevation in the low vitamin D group in a similar manner as Yilmaz and Şen [17] reported. We emphasise that the *P*-value of our comparison was in the border range (*P* = 0.07). We did not find any correlation between vitamin D level and CRP unlike the study of Daneshkhah *et al*. [21]. They observed that the CRP level was inversely correlated with 25(OH) vitamin D levels. We suggested that vitamin D may have a role in inflammation represented by CRP as a marker of the cytokine storm. Previous studies have shown that vitamin D modulates the release of inflammatory cytokines and chemokines [22, 23]. Some previous studies have found a negative correlation between vitamin D level and CRP level and increased production of proinflammatory cytokines, such as IL-6. Low vitamin D levels are also associated with increased inflammatory cytokine levels [24, 25]. Therefore, we suggest that vitamin D's immunomodulatory effect may be beneficial in COVID-19 infection. Hence, previous studies have supported the association between vitamin D status and clinical outcomes [25, 26]. As a result, vitamin D supplementation could provide a clinical benefit in critically ill patients with COVID-19. Vitamin D supplements to maintain the circulating 25(OH)D in the optimal levels (75–125 nmol/l) are recommended during this pandemic [27]. To date, however, no randomised controlled trial has evaluated the efficacy of vitamin D supplementation in COVID-19 patients.

This study has the following limitations. First, due to its retrospective design, some data, especially imaging studies (X-ray), were missing. Second, the patient group did not include patients with clinically severe disease because we did not treat critical patients at our centre. Third, we had to use local single-centre data limited to our hospital records.

## Conclusion

Our study provides an insight into the relationship between vitamin D deficiency and COVID-19 for future studies. Empiric intervention with vitamin D can be justified by its low serum levels. This suggests that vitamin D intervention, which is a safe and non-invasive treatment, may exert a potential benefit in reducing the severity of COVID-19 infection. The underlying mechanisms of vitamin D's role in the immunomodulation of COVID-19 warrant further investigation.

## Data Availability

The datasets used and/or analysed in the current study are available from the corresponding author on reasonable request.
